# Steroid-Induced Hyperglycaemia in a Patient With Possible Body Dissatisfaction: A Case Report

**DOI:** 10.7759/cureus.99776

**Published:** 2025-12-21

**Authors:** Chukwuebuka E Ohakpougwu, Wendy M Bebobru, Emmanuel Labram

**Affiliations:** 1 Psychiatry and Behavioral Sciences, Family Health University, Accra, GHA; 2 Psychiatry and Behavioral Sciences, School of Health Sciences Research, Chiang Mai University, Chiang Mai, THA; 3 Obstetrics and Gynecology, Lister Hospital, Accra, GHA; 4 Anatomical Sciences, Family Health University, Accra, GHA

**Keywords:** body dissatisfaction, effects of social media, side effects of social media, steroid abuse, steroid-induced hyperglycemia, young adolescent women, young people

## Abstract

Steroid use for body enhancement was historically associated with bodybuilding and skin bleaching, but now may be gaining traction as a means of non-surgical cosmetic enhancement for young women unsatisfied with parts of their body, such as the buttocks and hips. Here, the side effects of steroids, such as fluid retention and weight gain, are marketed as positive outcomes.

This case report describes a young African woman who presents with persistent hyperglycaemia, frequency, dysuria, and malaise after four months of taking dexamethasone tablets to gain a curvier frame. This case offers an opportunity to reassess our approach to history taking in young people presenting with hyperglycaemia and otherwise no family history of diabetes, especially in low- and middle-income countries (LMICs). Early identification of steroid abuse will enable further screening for comorbid mental health conditions, such as eating disorders and substance abuse, which are often associated with body dissatisfaction.

## Introduction

Body dissatisfaction can be defined as a negative attitude towards one’s own body, resulting from a discrepancy between actual body image perceptions and ideal body image [1,2]. This condition has been closely tied to eating disorders and social anxiety disorder and is more common in younger women with greater media consumption [3]. Individuals with body dissatisfaction have been shown to have a higher likelihood of using weight loss medications due to self-objectification and anti-fat attitudes by others [4]. Although there is a general trend towards using weight loss medication, weight gain - especially with the aim of achieving a curvier frame - is a growing trend among women in low- and middle-income countries (LMICs), with steroids being a popular choice since regulation of their over-the-counter sale is lax [5,6]. 

Steroids have been used extensively in almost every subspecialty of medicine, from short-term acute steroid therapy for chronic obstructive pulmonary disease (COPD) to foetal lung maturation [7]. However, beyond clinical use, steroids have found a home among other users, from bodybuilders and skin-toning/bleaching creams to their current role as a key medication in the non-surgical body enhancement market for women [8,9]. This market relies on selling a side effect of steroid abuse - fluid retention - as weight gain and enhancement of desired body parts, from the breasts and hips to the buttocks [5,10,11,12]. Unfortunately, steroids have been shown to lead to increased hyperglycaemia and, subsequently, diabetes by reducing insulin sensitivity and impairing the function of the glucose transporter type 4 (GLUT4), with a consequent reduction in insulin-stimulated glucose uptake [7,13,14]. Steroids also promote the deposition of fat in the viscera while reducing peripheral reserves, contributing to the accumulation of fat in muscle cells, with consequent interference in insulin signalling as well [15].

Numerous studies have related the use of steroids in body enhancement to body dysmorphia, and specifically to muscle dysmorphia in men [16]. New studies on steroid use in women have noted a radical shift in its use solely for conformity to new body standards of femininity, which emphasise less athletic builds and a curvier figure [17]. Social media influencers have contributed immensely to this trend, as they have increasingly marketed products for body enhancement, advancing narratives about beauty standards and gender stereotypes, especially to young people who might be highly impressionable [18]. In fact, a study in the United Arab Emirates directly linked extensive use of social media with negative appraisal of one’s physical appearance and a tendency to consider cosmetic enhancement [19].

The side effects of steroid use can be classified into immediate, gradual, and idiosyncratic. Fluid retention, weight gain, and insomnia are the immediate effects, whilst hyperglycaemia, osteopenia, dyslipidaemia, as well as central obesity and adrenal suppression, are the gradual side effects [15]. Finally, open-angle glaucoma, avascular necrosis, and psychosis are considered the idiosyncratic side effects of steroids [15].

Steroids are the main cause of drug-induced hyperglycaemia, and oral glucocorticoids have shown the highest prevalence, of up to 2%, in primary care populations [20,21]. A history of gestational diabetes and a family history of diabetes have been shown to increase one’s propensity to develop steroid-induced hyperglycaemia (SIHG) [4,15]. In addition, long-acting glucocorticoids, like dexamethasone, cause sustained hyperglycaemia lasting over 24 hours, as opposed to intermediate-acting steroids, like prednisone [14].

SIHG is defined as abnormally elevated blood glucose associated with the use of glucocorticoids in patients with or without pre-existing diabetes mellitus [22]. The diagnostic criteria for SIHG do not differ from other types of diabetes. These include a confirmed fasting blood glucose ≥7 mmol/L (≥126 mg/dL), a glucose level of ≥11.1 mmol/L (≥200 mg/dL) two hours following ingestion of 75 g glucose in an oral glucose tolerance test (OGTT), an HbA1c ≥6.5% (≥48 mmol/mol), or a random blood glucose ≥11.1 mmol/L (≥200 mg/dL) [23]. Even though the hyperglycaemic effect of steroids is believed to resolve once steroids are discontinued, this is not always the case, and studies have shown that between 10% and 50% of people with SIHG go on to develop type 2 diabetes [24]. 

SIHG among non-treatment-seeking individuals is currently understudied, and its presentation in this group might grow as the influence of fitness and health influencers spreads, especially among young people [25,26]. Thus, it is imperative that clinicians be prepared to identify this presentation early, manage it effectively, and coordinate care with mental health professionals to ensure prompt diagnosis of possible body dissatisfaction, eating disorders, and motivations that might compromise the patient’s health.

Hence, this case report presents a young patient who visited the clinic on account of poor glucose control after an oral course of dexamethasone taken for cosmetic reasons. This report aims to explore the presentation of SIHG in non-treatment-seeking patients, motivations behind steroid abuse, and management of SIHG, in a bid to sensitise clinicians.

## Case presentation

A 17-year-old presented to the Outpatient Department with a one-month history of increased fatiguability, frequency of urination, nocturia, polydipsia, and an abscess on her right thigh. She had been previously treated with ciprofloxacin 500 mg twice daily for one week at a peripheral hospital because she complained of lower urinary tract symptoms (frequency of urination, vaginal discharge, polyuria), for which a diagnosis of a urinary tract infection was made there. Two weeks after completing that medication, she presented to our facility because the lower urinary tract symptoms persisted. 

Upon thorough history taking, we determined that she felt unhappy with her current body image and wanted wider hips and larger buttocks. This was because she often felt unnoticed by the opposite sex at school and believed that enhancement of physical features was necessary. Hence, alongside six girls at school, she had begun to take oral dexamethasone (0.5 mg twice daily) after one of the girls in her friend group suggested it would help achieve her goals, based on information from a Facebook advertisement by an online vendor. The pills were subsequently purchased from this vendor and taken according to the vendor's prescription. Thus, she began to take the pill daily for the four months prior to presenting at our facility, and by the fourth month, she began to feel increasingly unwell, which prompted her visit to the peripheral hospital, where she was diagnosed with a urinary tract infection. At presentation to our clinic, she was still taking the pills, but now infrequently, as she was not sure if they were connected to her symptoms. We could not perform a psychometric assessment at the time because we did not have an in-house psychiatrist or psychologist. 

Furthermore, we found no history of diabetes in the patient, nor any family history of diabetes or other chronic medical conditions. Also, the patient had no past psychiatric history, nor any history of substance use or sexual abuse. The patient was a high schooler attending a boarding school at the time of presentation. She was the youngest of three children in her family and lived primarily with her mother.

On physical examination, the patient was not pale or anicteric and was stable on presentation with normal vital signs, but her BMI was in the range of overweight, as shown in Table 1. 

**Table 1:** Vital signs and BMI at presentation

Blood Pressure (mmHg)	Pulse (Beats per Minute)	Respiratory Rate (Cycles per Minute)	Weight (kg)	Height (cm)	BMI
116/70	90	22	60	66	138

She had an abscess on her right thigh, which she claimed began after sustaining a minor laceration while working at home. It is believed that her hyperglycaemia might have exacerbated poor healing of the initial wound, leading to abscess formation. She had a Ferriman-Gallwey score of 6 after assessment of hair distribution. Her random blood sugar on presentation was 31.8 mmol/L, and urine ketones were 3+. Hence, she was promptly admitted to the Emergency Department, and insulin perfusion was initiated to treat ketosis whilst pertinent laboratory tests were conducted; the results and reference ranges are shown in Table 2. Insulin infusion was given at 6 Units/hr, since the patient weighed 60 kg. Hourly monitoring ensued to ensure electrolytes were within the normal range and that the patient was not hypoglycaemic. She was also placed on 10% dextrose infusion at 125 mL/hr, as capillary glucose began to drop to 14 mmol/L and lower. Concurrently, the thigh abscess was drained, and a sterile dressing was applied. She was then placed on intravenous ceftriaxone 2 g daily while on admission.

**Table 2:** Laboratory tests done with references ranges and patient’s results Anti-GAD, anti-glutamic acid decarboxylase

Lab Test	HbA1C Normal (<5.7%), Prediabetic (5.5-6.4%), Diabetic (>6.5%)	White Blood Cell Count (4.0-11.0 x 10^9^/L)	Neutrophils (2.5-7.0 x 10^9^/L)	Urine Ketones (Negative or Trace)	Random Blood Glucose (3.9-7.8 mmol/L)	Fasting C-peptide (0.5-2.0 ng/mL)	Anti-GAD Antibody (Negative: 0-4.9 U/mL, Low Positive: 5-25 U/mL, Positive: >25 U/mL)	IA-2 Antibody (<10 U/mL)
Patient’s Result	9.3%	10.1	11.0	3+	31.8	1.2	<5	<10

After reviewing the patient's history, physical examination, and laboratory results shown in Table 2, a provisional diagnosis of SIHG was made following an endocrinologist consultation. Her anti-glutamic acid decarboxylase (anti-GAD) and fasting C-peptide were normal, which helped rule out type 1 diabetes. However, her glycated haemoglobin was 9.3%, suggestive of diabetes. By the third day of admission, the patient's fasting blood glucose remained steady between 11 and 12 mmol/L, and random blood glucose was repeatedly in the range of 13-15 mmol/L. The patient was then provided with nutrition counselling by an in-house dietician regarding the relationship between food, hyperglycaemia, and blood glucose control. Her mother was also given a diet plan to ensure strict adherence. 

Subsequently, the Mixtard insulin regimen (16 IU in the morning and 10 IU at night) was initiated, and insulin perfusion was discontinued. The patient was discharged after six days of admission. At discharge, she had a random glucose of 13 mmol/L and was asked to continue the current dose of Mixtard, 16 IU mane and 10 IU nocte. Her review was scheduled exactly one week after discharge. She and her mother were tasked with checking and recording her fasting and random blood glucose, which were to be reported at the first review. She was also taught the signs of hypoglycaemia and first aid for managing it. At discharge, the patient was given a referral letter to see a psychiatrist to explore reasons for possible body dissatisfaction, and a letter was written to accommodate her dietary needs at school. Furthermore, she went home on oral cefuroxime 500 mg bd for the abscess. Her mother declined attempts by physicians to contact her school, as she was worried that her daughter would be stigmatised or expelled, and refused to share any school contacts.

At the first review, she complained of recurrent episodes of light-headedness and recorded lower levels of fasting glucose (sometimes dropping to as low as 3.4 mmol/L), while random glucose was within the range of 10-13 mmol/L (Table 3).

**Table 3:** Table of random blood glucose at discharge and at first review

Time	Random Blood Glucose (mmol/L)
At Discharge	13-15
First Review	10-13

Thus, her insulin regimen was changed to Mixtard, 12 IU mane and 10 IU nocte daily. The patient was then scheduled to come for another review in a week’s time.

Unfortunately, there was no record of the patient presenting for the second review. When her mother was contacted via phone, she claimed the patient was doing okay but could not bring her on the scheduled date because of work. At the time of writing this report, there had been no new reviews. 

## Discussion

**Figure 1:**
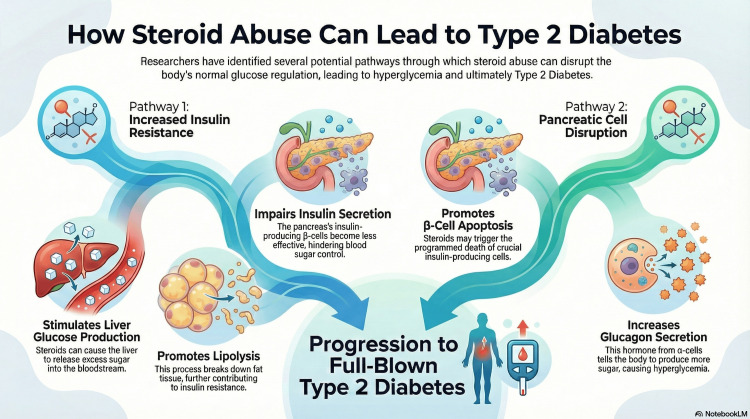
This diagram demonstrates the mechanisms of steroid impact on glucose metabolism leading to hyperglycaemia and subsequently type 2 diabetes Image generated using NoteBookLM (Google, Inc., Mountain View, CA, USA).

Currently, there is no generally accepted mechanism through which initial hyperglycaemia from steroid abuse can transform into type 2 diabetes, but there are several potential pathways. Some researchers conclude that steroids stimulate hepatic glucose production and lipolysis in adipose tissue, leading to increased insulin resistance and impairment of insulin secretion from β-cells in the pancreas [27]. Others believe that steroids promote β-cell apoptosis and increase glucagon secretion from α-cells, leading to persistent hyperglycaemia [27]. These mechanisms, portrayed in Figure 1, could be responsible for the patient having a glycated haemoglobin of 9.3%, which is diagnostic of diabetes.

This case report highlights a growing trend and craze for butt and hip enlargement driven by social media-endorsed standards of body image (curvier/hourglass shapes, as well as Brazilian Butt Lifts (BBLs)) [28]. Whilst many might shy away from surgery, filler injections, butt enhancement creams, and steroid pills are emerging as less invasive alternatives [28,29]. As social media use has become increasingly democratised, users have become younger and more impressionable. This can have negative consequences, including the adoption of unrealistic body standards and the rise of psychological and physical health complications, such as eating disorders, body dysmorphia, diabetes, and other cardiometabolic disorders [30]. Although it was particularly challenging to find literature on steroid use for buttock and hip augmentation, studies involving African women were identified and showed a gravitation towards using such medications prescribed at pharmacies, as well as herbal oral and topical medicines, to achieve desired results [28-31].

Glucocorticoids account for 2% of medication-induced diabetes globally and 56% of cases among hospitalised patients [32]. Diagnosing SIHG, especially when long-acting glucocorticoids are involved, is best done using post-prandial glucose and/or HbA1c [33]. No therapeutic goals are generally accepted, but some studies recommend a target of 7.8-10.0 mmol/L for hospitalised patients and 6.1-7.8 mmol/L for selected patients, whilst strictly monitoring for hypoglycaemia [34]. Monitoring for hypoglycaemia and educating patients on the associated symptoms can be lifesaving when initiating treatment for SIHG, as even the patient in this case had recurrent hypoglycaemic episodes, and her insulin dose had to be reduced.

Glucose-lowering therapy is recommended when pre- or post-prandial glucose repeatedly exceeds 7.8 or 11.1 mmol/L, respectively [22,34,35]. Oral hypoglycaemic agents can be recommended for inpatients who are stable, with non-critical disease and mild hyperglycaemia, but insulin is the mainstay of treatment [34,36]. Nevertheless, the type of insulin and the associated regimen used might depend largely on the type of glucocorticoid and the dose taken. For short-acting glucocorticoids, such as hydrocortisone, which induce hyperglycaemia acutely and often see a similar resolution, the decision to give insulin is made on a case-by-case basis [34]. However, rapid-acting insulin is often used, if needed, when hydrocortisone is present, and initiation of the dose is recommended at 0.1 IU/kg body weight [37]. Intermediate-acting glucocorticoids, such as prednisolone, require the use of intermediate-acting basal insulins, such as insulin detemir or neutral protamine insulin [34]. Some studies have recommended starting the insulin dose at 0.4 IU/kg of neutral protamine Hagedorn (NPH) insulin, whereas others have shown insulin glargine 100 IU, at a fixed starting dose of 0.5 IU/kg, to be equally effective in managing hyperglycaemia [38,39]. For long-acting glucocorticoids, such as dexamethasone, intermediate-acting basal insulin is recommended to be prescribed at an initial dose of 0.3 IU/kg body weight, but long- or ultra-long-acting basal insulin analogues, such as glargine, are also important alternatives [34]. Here, the dose is titrated downwards according to morning or evening glucose levels as follows: a reduction by 20% or 10% if levels fall below 4.1 mmol/L or between 4.1 and 6.0 mmol/L, respectively [40]. On the other hand, the dose is titrated upwards by 20% or 10% if glucose exceeds 18 mmol/L or is between 12.1 and 18 mmol/L, respectively [40]. Figure 1 aptly summarises the use of glucose-lowering agents in SIHG. In this case, the endocrinologist utilised Mixtard insulin, which is 70% intermediate-acting insulin, and titrated it downwards as blood glucose levels reduced and the patient exhibited signs of hypoglycaemia. 

It is important to note that a hyperglycaemic state might persist after stopping steroids and subsequently evolve into type 2 diabetes. Hence, patients need adequate education on SIHG, symptoms of hypo- and hyperglycaemic states, use of insulin pens, self-monitoring of blood glucose, and frequency of monitoring, whilst planning for HbA1c measurement every three months [37,41-43].

From our experience, a diagnosis of diabetes in young people can be challenging for caregivers, especially when injectable medication is prescribed. In our setting, seeking alternative treatment by family members to achieve faster resolution of symptoms is common, and this can often disrupt continuity of care. 

## Conclusions

SIHG, especially among young people in LMICs, is still under-researched and might emerge as a public health challenge, as more young people increasingly have access to social media, which might distort or negatively influence their perception of their bodies. Hence, it is crucial for clinicians to thoroughly explore the possibility of steroid abuse in young people presenting with hyperglycaemia, especially young women. It is also important to identify motivations for steroid abuse and provide mental health care as soon as possible, because of possible comorbid mental health conditions, such as eating disorders and body dissatisfaction. It is hoped that clinicians imbibe the art of thorough history-taking, so as not to miss the root causes of certain presenting complaints. 
